# Combination of Peglated-H1/HGFK1 Nanoparticles and TAE in the Treatment of Hepatocellular Carcinoma

**DOI:** 10.1007/s12010-022-04153-7

**Published:** 2022-09-12

**Authors:** Dazhi Gao, Xiangxian Xu, Ling Liu, Li Liu, Xiang Zhang, Xianxian Liang, Lanqi Cen, Qian Liu, Xiaoli Yuan, Zhenghong Yu

**Affiliations:** 1 The First Clinical Medical College of Nanjing University of Chinese Medicine, Nanjing, 210029 China; 2 Department of Interventional Therapy, Jinling Hospital Affiliated to Nanjing University, Nanjing, 210002 China; 3 Department of Radiology, Jiangsu Province Hospital of Chinese Medicine, Affiliated Hospital of Nanjing University of Chinese Medicine, Nanjing, 210029 China; 4 Key Laboratory of New Drug Research and Clinical Pharmacy, Xuzhou Medical University, Xuzhou, 221004 China; 5 Medical Imaging College, Xuzhou Medical University, Xuzhou, 221004 China; 6 School of Basic Medicine and Clinical Pharmacy, China Pharmaceutical University, Nanjing, 210009 China; 7 Department of Pharmacy, Xuzhou Infectious Disease Hospital, Xuzhou, 221004 China; 8 Department of Psychiatry, Jinling Hospital Affiliated to Nanjing University, No. 305 Zhongshan East Road, Nanjing, 210002 Jiangsu Province China; 9 Department of Oncology, Jinling Hospital Affiliated to Nanjing University, No. 305 Zhongshan East Road, Nanjing, 210029 Jiangsu Province China

**Keywords:** Transarterial embolization, Nanoparticles, PH1/HGFK1, Combination therapy, Hepatocellular carcinoma

## Abstract

Transarterial embolization (TAE) constitutes the gold standard for the treatment of hepatocellular carcinoma. The effect of combination of TAE and peglated-H1/HGFK1 nanoparticles was explored on hepatocellular carcinoma. MTT and Annexin V-FITC were used to determine the cell viability and apoptosis of HepG2, ml-1, LO2, and VX2 cells after the treatment of HGFK1. Next, the orthotopic rabbit was selected to establish the in situ models of VX2 hepatocellular carcinoma. Nanoparticles were synthesized with peglated-PH1 and used to deliver HGFK1 overexpressing plasmids. MRI was performed to monitor tumor volume after being treated with TAE. The protein expression levels of CD31, CD90, and Ki67 were determined by immunohistochemistry. H&E and TUNEL staining were used to determine the necrosis and apoptosis in vivo. HGFK1 significantly inhibited the proliferation and increased the apoptosis of HepG2 and ml-1 cells (*P* < 0.05). MRI on 14 days after modeling suggested that the tumor showed ring enhancement. MRI on 7 days and 14 days after interventional therapy showed that tumor volume was significantly inhibited after the treatment with TAE and HGFK1 (*P* < 0.05). The immunohistochemical results 7 days after interventional therapy indicated that the expressions of CD31, CD90, and Ki67 were significantly lower after treatment with TAE and HGFK1 (*P* < 0.05). TAE and HGFK1 all extended the survival period of rabbits (*P* < 0.05). PH1/HGFK1 nanoparticle is an innovative and effective embolic agent, which could limit angiogenesis post-TAE treatment. The combination of TAE with PH1/HGFK1 is a promising strategy and might alter the way that surgeons manage hepatocellular carcinoma (HCC).

**Supplementary Information:**

The online version contains supplementary material available at 10.1007/s12010-022-04153-7.

## Introduction

Hepatocellular carcinoma (HC) is the most common cause of cancer-related death, and the annual incidence rate may reach 1 million cases soon [1, 2]. Currently, transarterial embolization (TAE) constitutes the gold standard for the treatment of HC, especially for intermediate stage HC. TAE can block the blood supply of artery of tumor nodules and induce local ischemia and hypoxia, eventually leading to necrocytosis. Typically, injections of chemotherapeutic drug emulsions containing lipiodol are used in TAE to obtain synergistic effects of drug cytotoxic activity and ischemia. However, high recurrence and metastasis are still inevitable after TAE treatment. Recent accumulating evidence has shown that the expression of hypoxia-inducible factor-1α (HIF-1α) would be activated under the hypoxic-ischemic condition after TAE treatment, which could promote the expression of vascular endothelial growth factor (VEGF) and initiate angiogenesis, finally resulting in treatment failure [3]. Therefore, suppression of VEGF-mediated angiogenesis might be a promising strategy against HC. However, hypoxia induced by TAE might activate the HIF-α/VEGF signaling pathway.

The kringle 1 domain of human HGF a-chain (HGFK1, residues 123–210 of HGF) has been identified as an angiogenic inhibitor, with considerable anti-tumor activity in multiple tumors. H1-formed nanoparticle was easy to be blocked by reticuloendothelial system (RES) of body in case of systemic administration. Yao et al. have reported that HGFK1 could inhibit bone metastasis of breast cancer by activating the p38 mitogen-activated protein kinase (p38/MAPK) pathway [4]. In colon cancer, the combination of HGFK1 and recombinant adenovirus carrying p53 gene might suppress tumor angiogenesis via inhibiting the phosphorylation of epidermal growth factor receptor (EGFR) [5]. Our recent study also shows that HGFK1 attenuates angiogenesis and proliferation of HC through regulating EGFR and fibroblast growth factor (bFGF) signaling [6]. Similarly, Sun et al. find that HGFK1 may suppress neovascularization through regulating VEGF signaling pathway [7, 8]. Besides, the mesenchymal-to-epithelial transition (MET) signaling pathway, an important cancer cell “stemness” regulator, could be inhibited by HGFK1 in glioblastoma [9]. Hence, we suppose that HGFK1 possesses the potential to enhance the anti-tumor effect of TAE.

Adeno-associated viruses (AAV) vector has been widely used to encode target genes with its high transfection efficiency, which represents a significant tool to explore the gene function in vivo or in vitro [10]. Nevertheless, numerous challenges have limited its application in cancer, including cytotoxicity, neutralizing antibody responses, tissue transport, and infection of resistant cell types [11]. In our previous studies, we have developed a novel cationic copolymer consisting of low molecular weight polyethyleneimine (PEI, 600 Da), which is linked by β-cyclodextrin and conjugated with folic acid. It is named H1, which is characterized by low toxicity, highly effective, and biodegradable [12, 13]. Moreover, we have further developed Peglated-H1(PH1) by mixing H1 with polyethylene glycol (PEG, M.W.3350)-grafted polyethyleneimine-β-cyclodextrin (PEI-CyD) to avoid the clearance mediated by the reticuloendothelial system. PH1 has been documented as a safe and effective gene vector in vivo [14]. In this study, we investigated the anti-tumor effect of PH1/HGFK1 as an embolic agent for TAE in orthotopic rabbit HC models.

## Materials and Methods

### Cell Culture

The HC cell line HepG2, mouse HC cell line ML-1, rabbit squamous cell carcinoma cell line VX-2, and human fetal hepatocyte LO_2_ cells were purchased from American Type Culture Collection (ATCC, USA). The cells were cultured in Dulbecco’s modified Eagle medium (DMEM, Thermo Fisher, MA, USA) supplemented with 10% fetal bovine serum (FBS, Gibco, USA), 100 IU/ml penicillin, and streptomycin (Gibco, USA), and maintained in a humidified incubator with 5% CO_2_ at 37 °C.

### Preparation of PH1/HGFK1 Nanoparticles

The PEG3350-PEI600-CyD backbone was synthesized according to our previous study [13, 14]. In brief, polyethylene glycol (PEG3350, 250 mg) was dissolved in dimethyl sulfoxide (DMSO) and mixed with 1,10-carbon-yldiimidazole (CDI, 40 mg) under nitrogen atmosphere. The mixture was then added to the phosphate buffer solution (PBS) containing PEI600-CyD (240 mg) to obtain PEG3350-PEI600-CyD powder. Finally, H1 was mixed with PEG3350-PEI600-CyD at a molar ratio of 1:1 to obtain Peglated-H1 (PH1). To prepare PH1/HGFK1 polyplexes, PH1 and HGFK1 plasmids were mixed at an N/P ratio of 20:1 in the same volume. The polyplexes were stabilized for 10 min at room temperature before injection.

### MTT Assay

The cells were seeded on 96-well plates at a density of 1 × 10^4^ cells/well. Cells were treated with HGFK1 at 0, 5, 10, 20, 40, and 60 µg/ml for 48 h, respectively. Cell viability was assessed by MTT commercial kit (cat. #M6494, Invitrogen, USA) as the manufacturer described. The optical density of cells was measured by an automatic microplate spectrophotometer (SpectraMax 190, Sunnyvale, USA) at the absorbance of 595 nm.

### Flow Cytometry

The cells were seeded on 6-well plates at a density of 1 × 10^6^ cells/well. After overnight incubation, HepG2 and ML-1 cells were treated with 10 and 20 mg/ml HGFK1 for 48 h, respectively. Next, the cells were harvested in 500 µl 1 × binding buffer and incubated with 5 µl Annexin V-FITC and 5 µl propidium iodide (PI) for 15 min in the dark at room temperature. Finally, a flow cytometer (BD Biosciences, USA) was used to detect cellular apoptosis. The data were analyzed by FlowJo software.

### Animal Model

The study protocols were approved by the ethics committee of Jinling Hospital affiliated with Nanjing University. All experiments were carried out in accordance with ARRIVE (Animal Research: Reporting of In Vivo Experiments) guidelines. Forty-two New Zealand rabbits (weighted 2.0–2.8 kg) were purchased from the Model Animal Research Center of Nanjing University (SCXK(Su)2017–0011) and given free access to standard rodent food and tap water. The rabbit was subcutaneously inoculated in the left leg with VX-2 cells to form a solid tumor. Then the tumor tissues were cut into 27 mm^3^ pieces. After intravenous anesthesia, the abdominal cavity was opened to expose the liver. Sterile gauze soaked with normal saline was used to pull the left lobe of the liver. Ophthalmic scissor was used to make a 1-cm deep tunnel at the thickest part of the liver. Finally, the small tumor tissue was transplanted into the tunnel, and biological glue was used to oppress for 5 min. Subsequently, the abdominal cavity was closed. The rabbits were treated with antibiotics for 3 days postoperatively.

### Treatment with PH1/HGFK1 and TAE

Rabbits were randomly divided into four groups, including the control group, PH1/HGFK1 group, TAE group, and PH1/HGFK1 combined with the TAE group. The microcatheter was slowly delivered to the tumor vessel through curtails artery. For the control group, the rabbits received an administration of equal volume of PBS. For the PH1/HGFK1 group, the rabbits were administrated with 300 µg HGFK1 plasmids mixed with PH1. For the TAE group, 0.3–0.5 ml iodized oil was injected. For the PH1/HGKF1 + TAE group, the rabbits were administrated with HGFK1 plasmids and iodized oil in sequential order. After the microcatheter was removed, the blood supply was restored.

### Magnetic Resonance Imaging (MRI)

GE 3.0 T superconducting MRI instrument was used to measure the tumor before treatment. All rabbits were placed in a supine position for T1WI, T2WI, DWI, and DCE scanning after anesthesia. In axial position for T2WI, TR was 4800 ms, TE was 85.1 ms, the FOV was 15 cm, matrix was 160 × 256, NEX was 4, layer thickness was 3 mm, and 16 layers were scanned. In axial position for T1WI, TR was 520 ms, TE was 7.9 ms, FOV was 15 cm, the matrix was 128 × 128, NEX was 2, layer thickness was 3 mm, and 16 layers were scanned. DCE-MRI was performed using the liver acceleration volume acquisition (LAVA) sequence. TR was 2.7 ms, TE was 1.2 ms, FOV was 15 cm, the flip angle was 12°, matrix was 128 × 128, layer thickness was 3.2 mm, and 2000 layers were scanned. Contrast agent Gd-DTPA (0.5 mmol/kg) was injected in ear vein by the high-pressure injector. Before injection, they were scanned at 3°, 6°, 9°, 12°, and 15°. After injection of the contrast agent, 40 consecutive scans were performed within 160 s. The experimental rabbits were scanned again on days 7 and days 14 after treatment. Omni-Kinetics (GE Healthcare) was applied for measurement and analysis of MRI perfusion imaging data to obtain the value of *K*^trans^. The longest diameter (a) and shortest diameter (b) of tumor lesions were measured on T2 images, and the tumor volume was calculated according to the formula: *V* = *a* × *b*^2^/2. Besides, tumor status was assessed using mRECIST evaluation criteria [15].

### Hematoxylin–Eosin (HE) Staining and Immunohistochemistry (IHC)

Tumor tissues were fixed overnight in 4% paraformaldehyde for paraffin-embedded. The tissues were then sectioned 5-µm thick. For HE staining, the sections were stained with the commercial HE kit (cat. #C0105M, Beyotime, Shanghai, China) according to the manufacturer’s instructions. For IHC staining, the sections were deparaffinized, rehydrated, and boiled. After blocking the nonspecific antigen, the sections were incubated with the primary antibodies against CD90 (1:1000, cat. #13,801, Beverly, MA, USA), CD31 (1:2000, cat. # ab28364, Abcam, Cambridge, UK), and Ki-67 (1:2000, cat. # ab15580, Abcam, Cambridge, UK) overnight at 4 °C. The sections were then incubated with the secondary antibodies (1:2000, Abcam, Cambridge, UK) for 2 h at room temperature, followed by treatment with diaminobenzidine solution (DAB) (cat. #34,002, Thermo, USA) for 5 min. Images were acquired by using an optical microscope and analyzed by ImageJ software (National Institutes of Health, Bethesda, MD).

### TdT-Mediated Nick End Labeling (TUNEL)

Apoptosis in liver tissue was detected by TUNEL staining (cat. #11,684,817,910, Roche, Mannheim, Germany) according to the manufacturer’s protocol. Finally, nuclei were stained by hematoxylin. Images were acquired by using an optical microscope (OLYMPUS BX41, OLYMPUS, Tokyo, Japan) and photographed at 100 × magnification.

### Statistical Analysis

All data were analyzed using SPSS 22.0 software (SPSS, Inc, Chicago, IL, USA) and presented as means ± standard deviation (SD). The comparison among multiple groups was conducted by one-way ANOVA, while comparison between the two groups was analyzed by *t* test. Survival curves were plotted according to the Kaplan–Meier method, and the statistical significance between groups was determined using the log-rank test. *P* < 0.05 was considered statistically significant.

## Results

### HGFK1 Inhibited HC Cell Proliferation and Promoted Apoptosis In Vitro

**Fig. 1:**
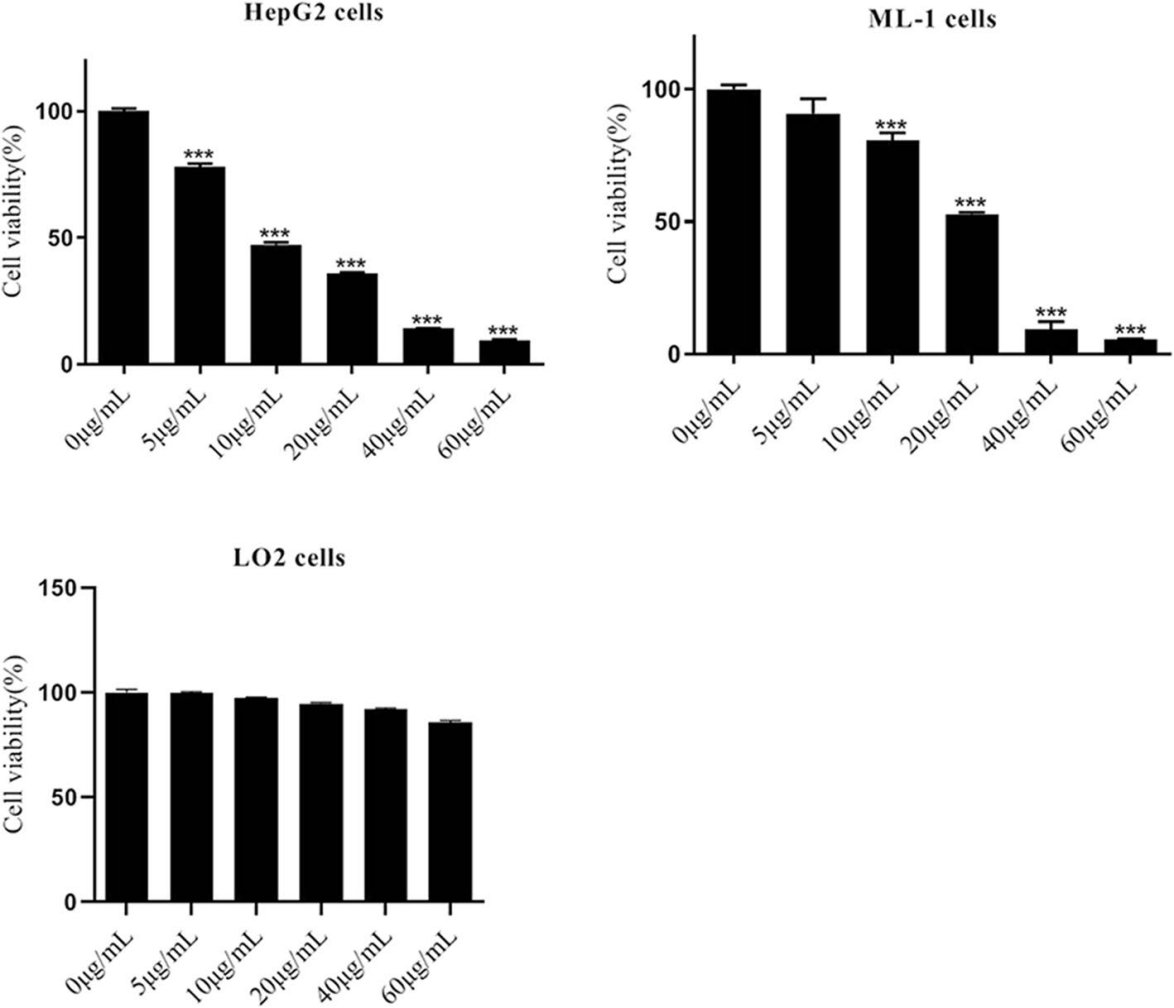
HGFK1 inhibited HC cell proliferation in vitro. MTT assay showed the cell viability of HepG2, ML-1, and LO2 cells treated with different doses of HGFK1 for 48 h. All data were presented as the mean ± SD from at least three independent experiments. *P < 0.05, **P < 0.01, ***P < 0.001 vs. the 0 µg/ml group

**Fig. 2:**
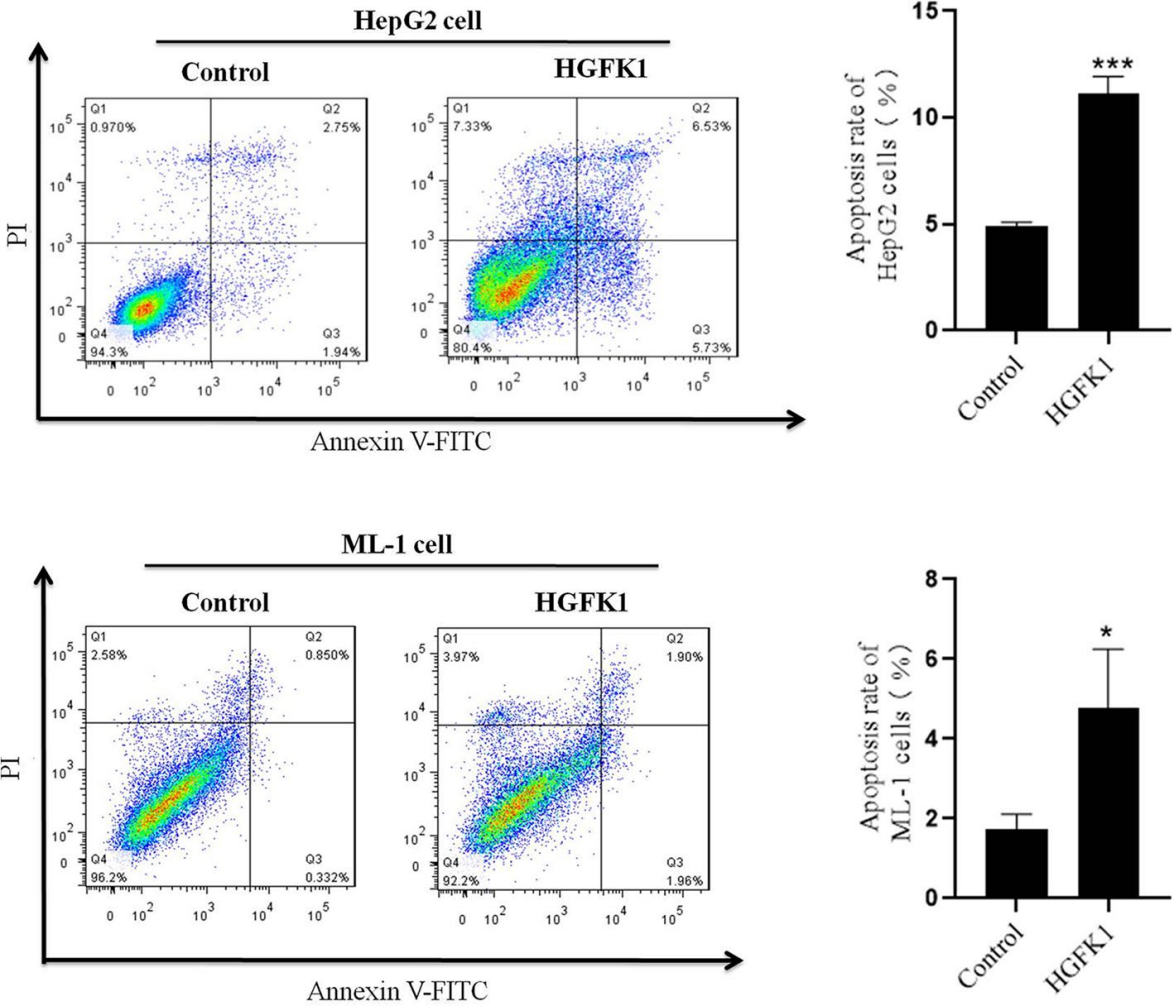
HGFK1 induced HC cell apoptosis in vitro. Representative images of flow cytometry analysis of HepG2 or ML-1 cells stained by Annexin V-FITC and PI, respectively. Quantification of cell apoptosis was shown for HGFK1 treatment in HepG2 or ML-1 cells, respectively. All data were presented as the mean ± SD from at least three independent experiments. *P < 0.05, **P < 0.01, ***P < 0.001 vs. the control group

To investigate the anti-tumor activity of HGFK1, we generated the purified recombinant HGFK1. The cell viability was significantly decreased in HepG2 and ML-1 cells after treatment with HGFK1 in a dose-dependent manner (*P* < 0.05). However, no significant change was observed in LO_2_ cells after treatment with HGFK1 (*P* > 0.05, Fig. 1). As shown in Fig. 2, flow cytometry showed that compared with the control group, the apoptotic rate of HepG2 cells was markedly increased in the HGFK1 group (4.91 ± 0.18% vs. 11.13 ± 0.8%, *P* < 0.001). Similarly, the apoptotic rates in the ML-1 cells were markedly increased about 2 folds in the HGFK1 group than in the control group (*P* < 0.05).

### The Combination of HGFK1 and TAE Prolonged the Survival Time

**Fig. 3:**
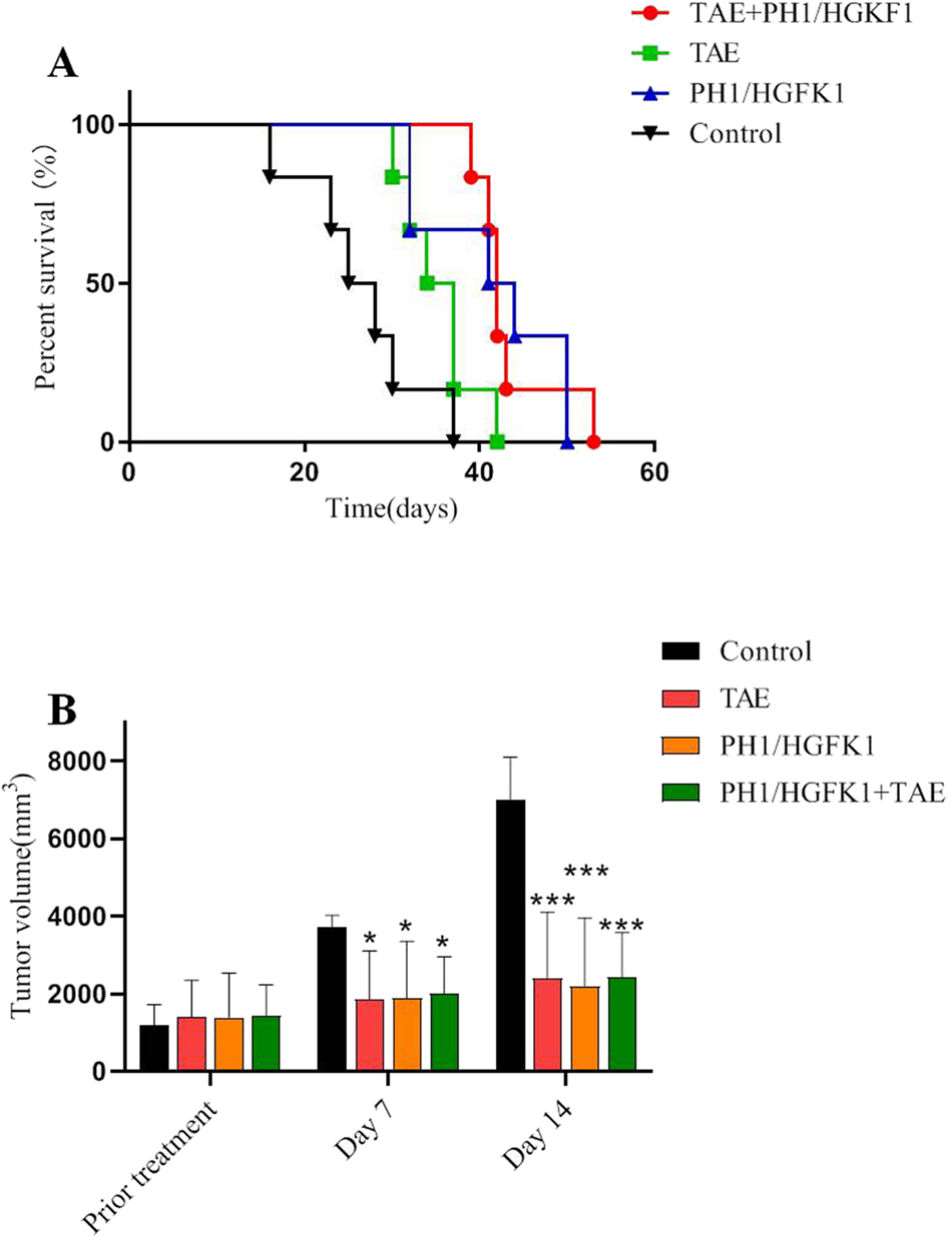
Tumor volume and survival rate of the xenografted rabbit model with different treatments. **A** Survival curve of tumor-bearing rabbits in control, PH1/HGKF1, PH1/HGKF1 + TAE, and TAE treated groups. **B** The dynamic change in tumor volume on prior treatment, day 7, and day 14 in control, PH1/HGKF1, PH1/HGKF1 + TAE, and TAE treated groups. All data were presented as the mean ± SD from at least three independent experiments. *P < 0.05, **P < 0.01, ***P < 0.001 vs. the control group

To evaluate the anti-tumor effect of PH1/HGFK*1* in vivo, we first established the rabbit HC orthotopic transplantation model with a success rate of 85.7% (36/42). As shown in Fig. 3A, the survival rate dropped to 0 in the control group on day 27, which was prolonged to 36 days in the TAE group. Furthermore, the survival time has increased to 42 days in the PH1/HGKF1 group and 43 days in the PH1/HGKF1 + TAE group (*P* > 0.05). When compared with the PH1/HGKF1 + TAE group, the mean survival time was significantly shorter in the control group and the TAE group (*P* < 0.05).

### The Combination of HGFK1 and TAE Inhibited Tumor Growth In Vivo

The tumor size on days 7 and 14 was all significantly smaller in PH1/HGKF1 + TAE group compared with the control group (*P* < 0.05). There was no significant difference in the tumor size between the PH1/HGKF1 and TAE groups (*P* > 0.05). Meanwhile, the tumor size was the smallest on day 14 in the PH1/HGKF1 group (Fig. 3B). After modeling, the tumor area showed a low signal on T1WI and a high signal on T2WI with a more obvious signal in the center of some tumors in all HC orthotropic transplantation rabbits. Moreover, DCE perfusion enhancement showed that there was no blood supply in the center of the tumor. There was no statistically significant difference in lesion size between the four groups before treatment. Seven days after treatment, the tumor enhancement area was significantly reduced in both PH1/HGKF1 + TAE group and the TAE group (Supplementary Fig. 1).

Previous studies have determined that the microvascular contrast agent transfer constant (*K*^trans^) plays an important role in tumor prognosis [16]. As shown in Table 1 and Supplementary Fig. 2, the *K*^trans^ values on days 7 and 14 were dramatically decreased in the three treatment groups in comparison with the control group (*P* < 0.05). However, the lowest *K*^trans^ level was found in the PH1/HGKF1 + TAE group (*P* < 0.05). Moreover, the *K*^trans^ value in the TAE group on day 14 was twice than that in the PH1/HGKF1 + TAE group (*P* < 0.05).

**Table 1:** Comparison of *K*^*trans*^ value among four groups

Groups	Cases	Pro-treatment (min^−1^)	Days 7 (min^−1^)	Days 14 (min^−1^)
PH1/HGKF1 + TAE	6	0.46 ± 0.11	0.23 ± 0.01**	0.12 ± 0.02**#
TAE	6	0.47 ± 0.03	0.32 ± 0.03*	0.25 ± 0.04*
PH1/HGKF1	6	0.49 ± 0.03	0.26 ± 0.04*	0.18 ± 0.06**
Control	6	0.42 ± 0.04	0.57 ± 0.05	0.64 ± 0.03

^*^P < 0.05, **P < 0.01, ***P < 0.001 vs. the control group; #*P* < 0.05, ##*P* < 0.01, ###*P* < 0.001 vs. the TAE group

### The Combination of HGFK1 and TAE Inhibited Proliferation and Angiogenesis and Induced Apoptosis In Vivo

**Fig. 4:**
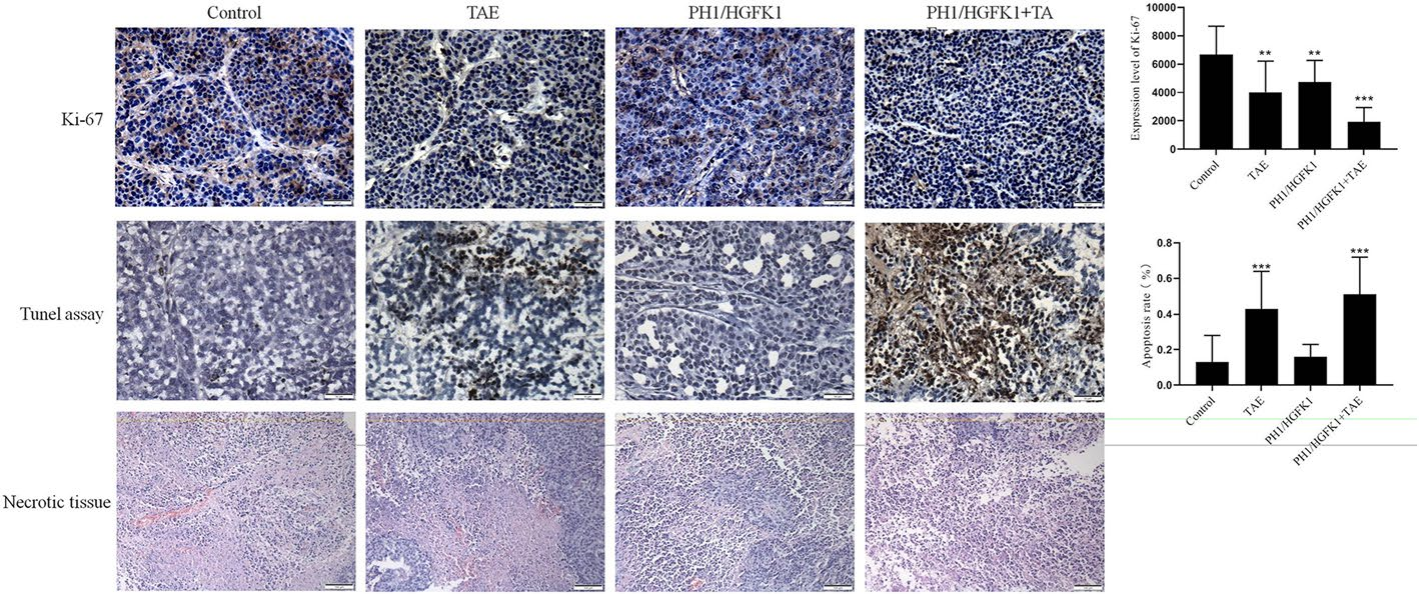
Effect of TAE and PH1/HGFK1 on tumor proliferation, apoptosis, and necrosis in vivo. Representative immunohistochemistry images and analysis of the Ki-67, TUNEL expression level in tumor tissues of control, PH1/HGKF1, PH1/HGKF1 + TAE, and TAE alone treated groups. Bar = 50 mm. Brown dots indicated Ki-67, TUNEL-positive signaling. Cell nuclei were stained by hematoxylin. Representative H&E images of tumor necrotic tissue in the rabbit model with different treatments. Bar = 50 mm. All data were presented as the mean ± SD from at least three independent experiments. *P < 0.05, **P < 0.01, ***P < 0.001 vs. the control group

**Fig. 5:**
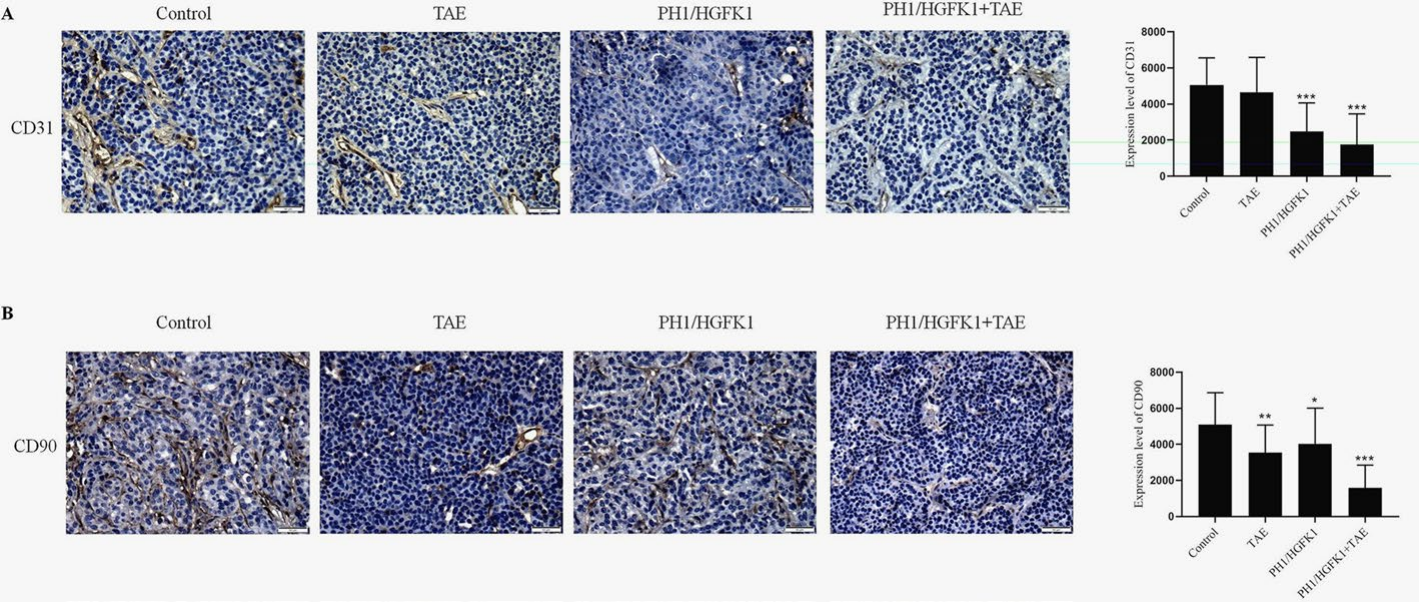
Effect of TAE and PH1/HGFK1 on the expression level of CD90 and CD31 in vivo. **A** Representative immunohistochemistry images and analysis of CD31 expression level in tumor tissues of control, PH1/HGKF1, PH1/HGKF1 + TAE, and TAE treated groups. Brown dots indicate CD31-positive signaling. Cell nuclei were stained by hematoxylin. Bar = 50 mm. **B** Representative IHC images and analysis of CD90 expression level in tumor tissues of control, PH1/HGKF1, PH1/HGKF1 + TAE, and TAE treated groups. Brown dots indicated CD90-positive signaling. Cell nuclei were stained by hematoxylin. Bar = 50 mm. All data were presented as the mean ± SD from at least three independent experiments. *P < 0.05, **P < 0.01, ***P < 0.001 vs. the control group

The lowest Ki-67 level and the highest apoptotic signal were observed in the PH1/HGKF1 + TAE group, while the highest Ki-67 expression and the lowest apoptotic signal were observed in the control group. Moreover, we found that PH1/HGKF1 transfection could not augment apoptosis in vivo. Besides, HE showed a bigger necrotic area in the PH1/HGKF1 + TAE and TAE groups than that in the control group (*P* < 0.05, Fig. 4). We further determined the expression of CD31 in tumor tissues by IHC. The results showed that the expression of CD31 was significantly decreased in the PH1/HGFK1 and PH1/HGKF1 + TAE groups in comparison with the control group (*P* < 0.001). Inversely, the expression of CD31 in the TAE group was similar with that in the control group (Fig. 5A).

### Combination of HGFK1 and TAE Inhibited Cancer Stem Cells in HC Rabbits

Increasing pieces of evidence suggested that drug resistance was associated with the maintenance of cancer stem cells (CSCs)-like phenotype [17, 18]. Our data showed that TAE and PH1/HGFK1 treatment could significantly reduce the expression of CD90 in the rabbit HC model (*P* < 0.05). Besides, in the PH1/HGFK1 + TAE group, the expression level of CD90 was significantly downregulated (*P* < 0.001) (Fig. 5B).

## Discussion

HCC ranks fifth in the incidence of cancer and third in the mortality rate worldwide. There are nearly 750,000 new cases worldwide every year, 90% of which are HCC [19]. China has become a high incidence area of HCC because of its high incidence rate of viral hepatitis. About 110,000 patients die of HCC every year, accounting for 45% of the global death toll of HCC [20]. For the treatment of liver cancer, more than 60% of patients undergo local, regional, or systemic palliative therapies [21]. Transvascular therapies are widely used for HCC, including transarterial embolization (TAE), conventional transarterial chemoembolization (cTACE), TACE with drug-eluting beads (DEB-TACE), and radioembolization (SIRT) [22]. Indeed, a combination of chemotherapeutic drugs has emerged as the mainstream operation method [23]. Of all the transvascular therapies, cTACE is the most popular one.

TACE is recommended as the first choice for the treatment of patients with stage B HCC in the Barcelona Clinic Liver Cancer (BCLC) stage and is widely used in the clinic. However, as a palliative treatment, TACE is still faced with a high rate of local recurrence. In recent years, some scholars put forward the concept of TACE resistance. TACE resistance or failure is defined as the same lesion is still in a progressive or stable state after more than 3 times of TACE treatment within 6 months. The reason may be related to the following factors [24–26]. (1) The HCC tissue has a dual blood supply, and the central area is mostly supplied by the hepatic artery, while the marginal area is mostly supplied by the portal vein. TACE alone has little effect on the peripheral blood supply area of the lesions, so the residual cancer cells can lead to recurrence. (2) Tumor tissue is in a state of ischemia and hypoxia after TACE, which stimulates liver cancer cells to secrete vascular endothelial growth factor (VEGFR), leading to revascularization, formation of collateral circulation around the lesion, and even recanalization of the original supplying artery. (3) The existence of tumor stem cells resistant to ischemia and hypoxia environment is also the reason for the poor efficacy after TACE.

Accumulating evidence has shown that Met is an independent surface marker of glioma stem cells [27]. Some scholars also suggest that Met may also be a specific surface marker of liver cancer stem cells [28]. Relevant studies have shown that the ischemia and hypoxia state after TACE and the effect of chemotherapeutic drugs can upregulate the expression of c-Met, a member of tyrosine kinase receptor family, and its ligand is hepatocyte growth factor (HGF) [29]. Abnormal activation of the HGF/c-Met signaling pathway is often accompanied by resistance against anti-tumor treatment and tumor metastasis. Human hepatocyte growth factor tricyclic fragment (HGFK1) is a domain of HGF, which has the effects of anti-angiogenesis and inhibiting tumor cell invasion and metastasis. Therefore, gene therapy for the c-Met signaling pathway has broad research prospects.

Gene therapy cannot continue to play an effective role in cells because it is easy to be degraded by ribozymes with the poor ability of endocytosis and endosome escape. In our previous study, a new method is synthesized by β-cyclodextrin crosslinked low molecular weight polyethyleneimine (PEI) grafted folate positron polymer (PEI-CyD-FA, named H1). The vector can concentrate and package the plasmid into spherical nanoparticles with a diameter of about 100 nm. Subsequent animal and cell experiments have confirmed that the vector has the characteristics of low toxicity, high transduction efficiency, and folate ligand targeting. PEG is coupled to H1 to form a new carrier, which is named PH1 after being mixed with H1 in equal proportion. HGFK1 is transduced to explore its anti-HCC effect.

The relevant results of our previous study show that HGFK1 could enhance the sensitivity of glioma cells to radiotherapy. In the mouse glioma in situ models, the intraperitoneal and peritumoral injection of nanoparticles transduced with HGFK1 could inhibit the growth of glioma and increase the sensitivity of glioma to radiotherapy. In addition, in the transplanted tumor model of mouse liver cancer, we found that by injecting PH1/HGFK1 through the tail vein, can significantly inhibit the growth of the tumor. There are significant statistical differences in survival time, immunohistochemical results, and changes of liver cancer stem cell markers compared with the normal saline. The combination with sorafenib can obtain better anti-HCC efficacy.

Thus, in this study, we proposed a novel strategy against HCC by combining HGFK1 and TAE. Our results have demonstrated that HGFK1 could repress the proliferation of multiple liver cancer cells and promote apoptosis in a dose-dependent manner, while it would not affect normal cell growth. The previous study has demonstrated that PH1 nanoparticle could effectively deliver drug with many advantages, including low cytotoxicity and highly effective, making it possible for gene therapy in vivo [14]. In this study, the results have shown that PH1/HGFK1 could significantly improve the survival time of tumor-bearing rabbits. And then, PH1/HGFK1 inhibits tumor cell growth and angiogenesis as well as the number of cancer stemness in vivo, indicating the positive anti-tumor role of HGFK1 in HCC.

After combination PH1/HGFK1 treatment with TAE, the tumor growth is suppressed, and the survival time is significantly prolonged compared with TAE treatment alone. The IHC results show that the expression of CD31 is dramatically reduced. Currently, patients with cancer with high CD31 expression show a poor prognosis [30]. Overexpression of CD31 could trigger aberrant angiogenesis through HIF-α in many types of tumors. Unfortunately, hypoxia induced by TAE might activate the HIF-α/VEGF signaling pathway [31]. Hence, a combination of PH1/HGFK1 and TAE might remedy this deficiency. Furthermore, the effect of TAE on apoptosis and cancer stemness is enhanced in the combined therapy.

Cancer stem cells are a small subpopulation within tumors with self-renewal capacity and multipotency, which involves tumorigenesis, metastasis, tumor recurrence, and drug resistance [32]. To evaluate this function, we have detected the stem-associated gene, CD90, in our study. The expression of CD90 is dramatically reduced in the PH1/HGFK1 + TAE group compared with TAE alone or PH1/HGFK1 alone. Our results suggest that TAE treatment alone reduces proliferation and upregulates apoptosis but does not suppress angiogenesis, while PH1/HGFK1 treatment alone inhibits tumor growth and angiogenesis but does not induce apoptosis. However, a combination of PH1/HGFK1 and TAE could simultaneously suppress tumor growth and angiogenesis in HCC. One limitation of this study is that the body weight, urine, and feces volume and excretion as well as the food intake during the treatments have not been recorded.

In conclusion, PH1/HGFK1 nanoparticle is an innovative and effective embolic agent, which could limit angiogenesis post-TAE treatment. The combination of TAE with PH1/HGFK1 is a promising strategy and might alter the way that surgeons manage HCC.

## Electronic supplementary material

Below is the link to the electronic supplementary material.

Supplementary file1 Representative MRI images of the rabbit model with different treatmentson prior treatment, day seven, and day 14. (jpg 1.63 MB)

Supplementary file2 Representative K^trans^ images of rabbit model with differenttreatments on prior treatment, day 7, and day 14. (jpg 2.31 MB)

## Data Availability

N/A.

## Abbreviations

*HC*: Hepatocellular carcinoma
*TAE*: Transarterial embolization
*HIF-1α*: Hypoxia-inducible factor-1α
*VEGF*: Vascular endothelial growth factor
*EGFR*: Epidermal growth factor receptor
*bFGF*: Fibroblast growth factor
*MET*: Mesenchymal-to-epithelial transition
*AAV*: Adenovirus-associated virus
*PBS*: Phosphate buffer solution
*PH1*: Peglated-H1
*PI*: Propidium iodide
*MRI*: Magnetic resonance imaging
*LAVA*: Liver acceleration volume acquisition
*HE*: Hematoxylin-eosin
*IHC*: Immunohistochemistry
*DAB*: Diaminobenzidine solution
*TUNEL*: TdT-mediated nick end labeling
*SD*: Standard deviation
*CSCs*: Cancer stem cells
*cTACE*: Conventional transarterial chemoembolization
*BCLC*: Barcelona Clinic Liver Cancer
*VEGFR*: Vascular endothelial growth factor
*HGFK1*: Hepatocyte growth factor tricyclic fragment
*PEI*: Polyethyleneimine
