# Supplementary figures and images for: Combination of Peglated-H1/HGFK1 Nanoparticles and TAE in the Treatment of Hepatocellular Carcinoma
Source: Appl Biochem Biotechnol. 2022 Sep 12;195(1):505–18. doi: 10.1007/s12010-022-04153-7 (PMC9832107; doi:10.1007/s12010-022-04153-7)

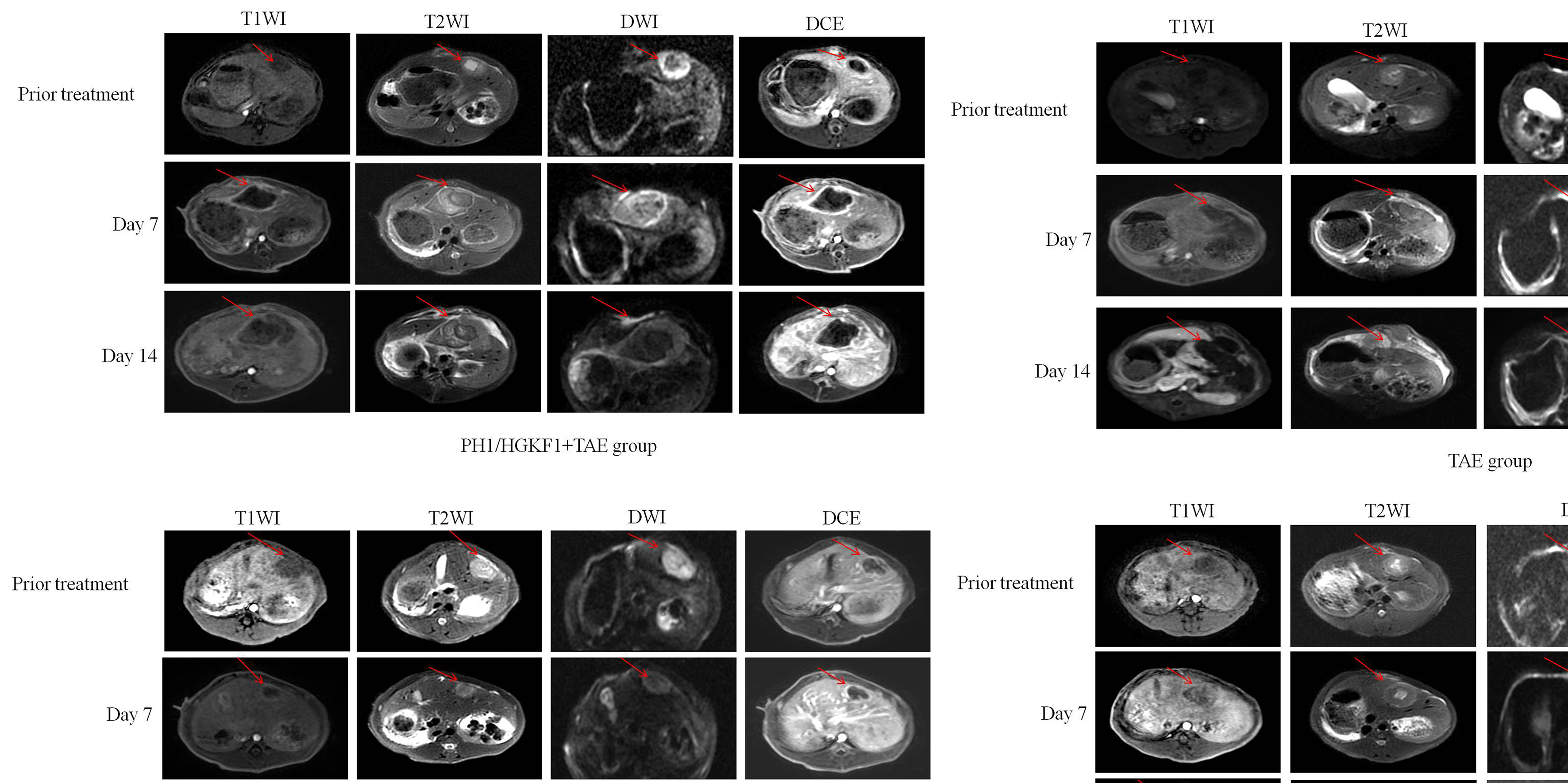

Supplement: Supplementary file 1 — Supplementary file1 Representative MRI images of the rabbit model with different treatmentson prior treatment, day seven, and day 14. (jpg 1.63 MB) [file 12010_2022_4153_MOESM1_ESM.jpg]

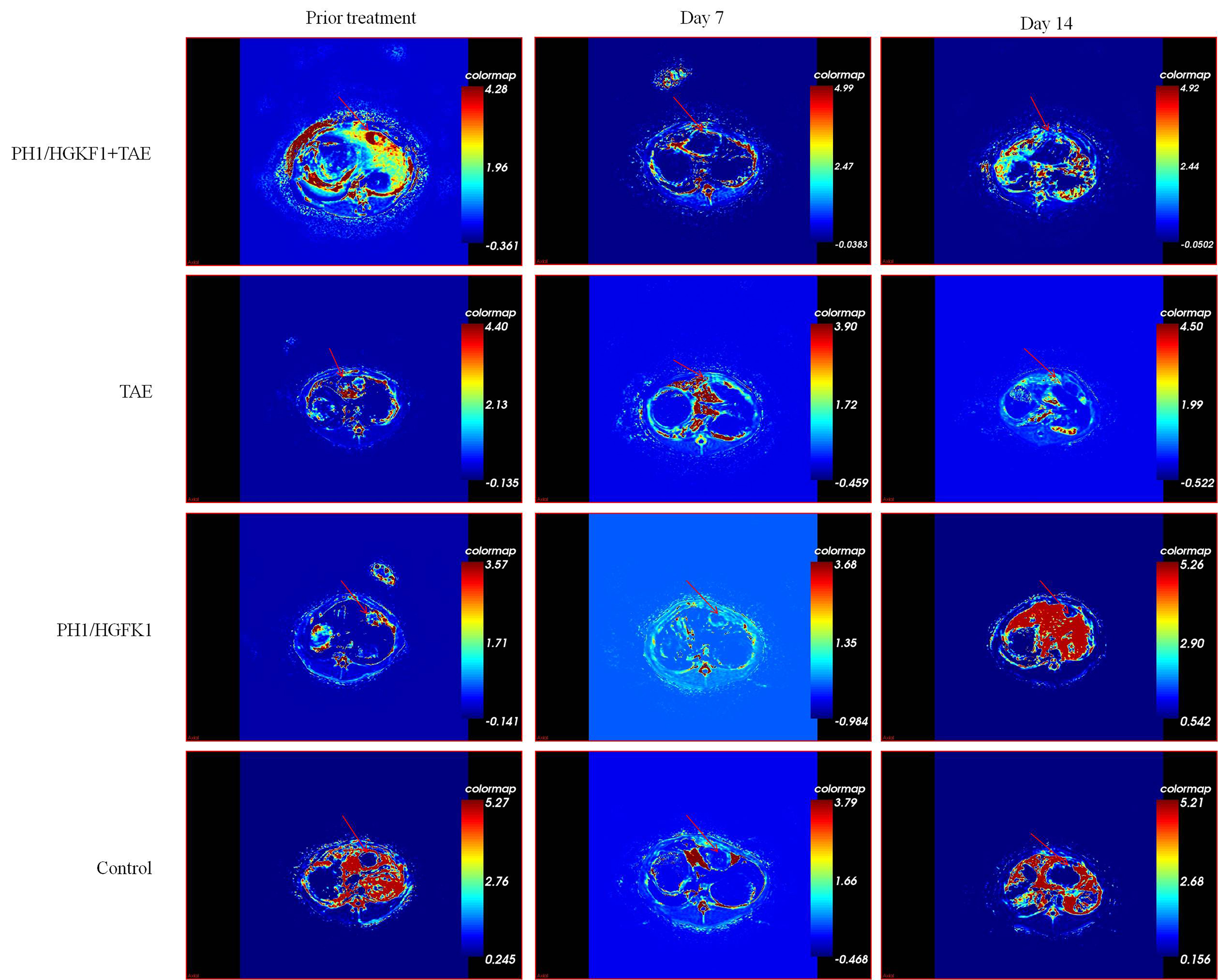

Supplement: Supplementary file 2 — Supplementary file2 Representative Ktrans images of rabbit model with differenttreatments on prior treatment, day 7, and day 14. (jpg 2.31 MB) [file 12010_2022_4153_MOESM2_ESM.jpg]
